# Horse owners seeking online health information: a mixed-methods study

**DOI:** 10.3389/fvets.2025.1628421

**Published:** 2025-07-23

**Authors:** Laura Haase, Julia Winter, Sophia Grummt, Martin Sedlmayr, Brita Sedlmayr

**Affiliations:** ^1^Carl Gustav Carus Faculty of Medicine, Institute for Medical Informatics and Biometry, Technische Universität Dresden, Dresden, Germany; ^2^Department of Cooperative Studies - Computer Science, Berlin School of Economics and Law, Berlin, Germany

**Keywords:** animal health information seeking, horse health information seeking, online health information seeking, proxy seeking, human computer interaction, animal owners

## Abstract

**Background:**

Many people seek health-related information online, not only for themselves but also on behalf of others who cannot articulate their symptoms. This proxy information-seeking behavior is particularly relevant for animal owners, who must interpret their animals’ symptoms without direct verbal feedback. While online health information-seeking in the context of one’s own health is well-studied, the specific challenges of searching by proxy, especially for animal health information, remain largely unexplored.

**Objective:**

This study aimed to determine the specific information needs and search behavior of animal owners. As a case study, horse owners were selected, representing a group regularly searching the web for health-related advice concerning their animals.

**Methods:**

A mixed-methods approach was used with 17 horse owners in Germany. Participants first described a recent search for equine health information. They were then shown a video of a horse experiencing an asthma attack and asked to conduct a search on how to proceed with the horse’s condition. Afterwards, they were questioned about their respective search behavior.

**Results:**

The participants’ main initial questions revolved around the cause of the horse’s condition, its urgency of veterinary treatment and the cost of treatment. All participants chose the Google search engine as the starting point for their search and formulated an average of 3.71 (SD: 2.02) queries. Each of these queries contained an average of 3.81 words (SD: 1.57). Most searches (52%) were evidence-directed with 29% using multiple descriptors of the horse’s situation. An average of 0.97 results (SD: 1.38) were clicked per query, with titles containing all search terms in 13% of cases. Participants reported experiencing several barriers to their search, including difficulties in formulating precise queries and the need for additional guidance during the search process.

**Conclusion:**

The findings highlight the need for improved online information systems, offering better guidance, context-aware search support, and trustworthy sources. The insights could inform veterinarians on how to better address their clients’ communication and information needs, provide them with the skills and knowledge they need to conduct online research and therefore build a better animal health partnership with them.

## 1 Introduction

The Internet has become a primary resource for individuals seeking health-related information, helping them reduce uncertainty, understand medical conditions, enhance self-care, and make informed decisions (1–3). This trend is particularly pronounced among younger populations, likely due to the ease of accessibility of online resources (2). In a study that monitored participants’ Internet usage over a three-month period, approximately two-thirds of the subjects conducted at least one online search on health-related information (1).

In some cases, individuals engage in health information proxy searching – seeking information on behalf of another individual who cannot articulate their condition. This is common, e.g., in parents and (pet) animal owners, whose searching behavior is possibly linked to their full responsibility for another individual’s well-being (4, 5). Although literature suggests a resemblance between both group’s proxy searches (6), unlike parents, animal owners must often navigate a more fragmented landscape of information, relying on veterinarians, online resources, and peer discussions (5). Additionally, compared to human healthcare, seeking online information for animal health presents unique challenges, including fundamental anatomical and physiological differences between species, and varying levels of veterinary literacy among owners (5). The decision-making process for animal health is further influenced by financial considerations, as veterinary care may not always be immediately affordable (7), because animals are not widely covered with health insurance (8).

Despite the increasing reliance on online searches for animal health concerns (9), little is known about how animal owners formulate search queries, evaluate sources, or navigate barriers to accurate information. Understanding these behaviors is essential not only for designing better information systems that provide reliable, actionable, and context-aware guidance for those seeking veterinary advice online, but also for improving veterinary-client communication.

**Figure 1:**
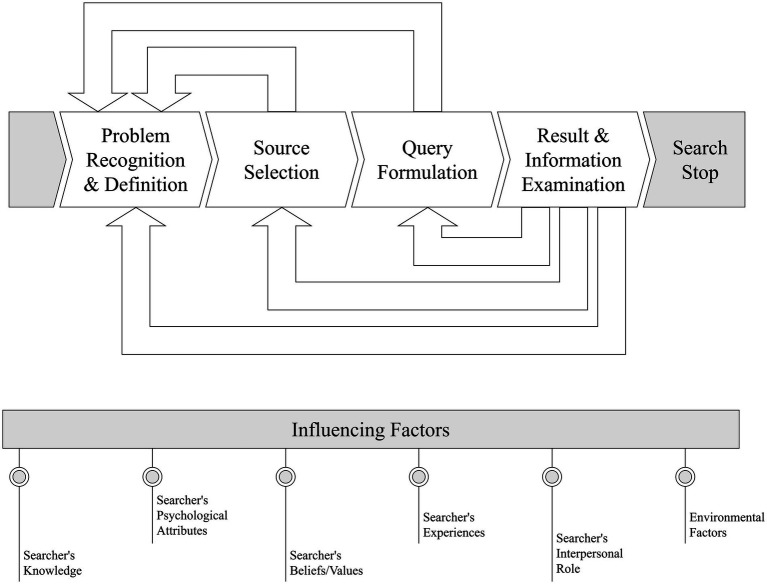
Summarized information-seeking process.

The information-seeking process typically begins with the recognition and definition of a problem (10–13), whereby an individual acknowledges a deficiency in their information necessary to address a specific question (1). Although the subsequent steps of the information-seeking process might vary in their exact representation in the literature, the process generally continues with the selection of an appropriate information source, the formulation of a search query, and the evaluation of the resulting information (10, 12–15). Each step may be repeated. Furthermore, information behavior is influenced by various factors, including the individual’s existing knowledge, psychological attributes, beliefs, values, and experiences, as well as their interpersonal role. Environmental factors, such as cultural influences and financial constraints, are also essential for shaping the information-seeking process (11–13) (Figure 1).

When individuals seek information regarding acute health symptoms, they generally engage in one or more of the following strategies: a symptom-directed search aimed at evidence gathering (evidence-directed search approach), a disease-directed search functioning as hypothesis testing (hypothesis-directed search approach), and/or a search for action recommendations or treatments (16). The search process typically concludes when the individual has obtained all the desired information from their mental list and achieved a stable mental model of the issue (17).

A key challenge for animal owners is deciding when veterinary consultation is necessary – a decision-making process where many seek additional guidance (5). While there are specialized online triage services (mostly video consultations) available [e.g., (18, 19)], these usually involve fees that animal owners may try to avoid. This study aims to identify the specific information needs of horse owners and analyze their online search behavior when seeking equine health information. By exploring these patterns, the study provides insights into how online information systems are used in everyday life.

## 2 Materials and methods

### 2.1 Study design

The study employed a mixed-methods design, incorporating both qualitative and quantitative components. It consisted of an initial interview about a previous online search related to equine health, a search task conducted during the study session, and a follow-up qualitative interview supplemented by quantitative questionnaires. The study personnel used a written study guide during the appointments to ensure consistency among all participants. The entire study was conducted in German. The study design was pre-tested twice to evaluate comprehensibility, sequence, feasibility, and time expenditure, which resulted in modifications to certain question formulations.

#### 2.1.1 Initial interview

After receiving a brief introduction to the aims and procedures of the study, participants were asked to describe a recent search for information on the acute health of one of the horses they owned. They were also asked to describe the situation of this search and the search device they used.

#### 2.1.2 Search task

For the search task, participants were instructed to imagine being at their horse’s stable late in the evening, where they encountered a scenario depicted in a brief video showing a horse experiencing an asthma attack. After watching the video, participants were asked to articulate any thoughts or questions that arose from the situation. They were then directed to use the Internet to determine how to proceed with the horse’s condition, conducting the search as they would in a real-life scenario while verbalizing their thought processes (thinking-out loud). Participants were encouraged to ask any clarifying questions about the horse’s condition they would be able to check with their horse in a real-life situation. When asking questions, participants were provided with all available information corresponding to the scenario by the study personnel. The video could be re-watched as often as necessary, including during the search. The study personnel refrained from intervening during the search process, except when participants ceased to express their thoughts aloud. In instances where participants indicated that they would immediately contact their veterinarian, they were instructed to assume a scenario in which the veterinarian was not immediately accessible.

#### 2.1.3 Follow-up interview and questionnaires

Upon indicating that they wished to conclude the Internet search (with no imposed time constraints by the study personnel), participants were prompted to complete an online survey. This survey included items on the satisfaction with the search process, the search results, objectives, and subsequent intended actions, the perceived alignment between their search behavior during the study and their every-day search behavior, factors that either facilitated or impeded the search, as well as follow-up questions on the think-aloud procedure (if applicable). Subsequently, participants completed questionnaires measuring their attachment to their horse [using a translated and adapted version of the Lexington Attachment to Pets Scale, LAPS (20)] and their health anxiety concerning their horse [using an adapted version of the modified Short Health Anxiety Inventory, mSHAI (21)]. Alongside those surveys, they were asked about the duration of their horse ownership, their participation in equestrian disciplines and competitions, the frequency of experienced horse illnesses, and their self-assessed knowledge of equine health. Finally, participants were given the opportunity to provide any additional comments regarding the study.

Quantitative items were answered directly within the online survey, with the possibility to verbally elaborate on the responses if desired, while qualitative questions were answered directly verbally.

#### 2.1.4 Study appointments

Study appointments were conducted in person at a date, time, and location that best accommodated the participant’s schedule, provided that the site offered a quiet environment and an Internet connection. Each session was carried out on a one-to-one basis with study personnel. To ensure a consistent technical setup, study personnel provided a laptop equipped with a standard mouse and a keyboard for the search task. Both the audio and the laptop screen were recorded to facilitate subsequent analysis.

### 2.2 Recruitment

Participants were recruited through personal networks and social media of the authors, with recipients encouraged to further disseminate the flyer with study information and call for participation.

Inclusion criteria were: (1) current ownership of at least one horse, (2) having conducted at least one Internet search related to the health of one of their horses within the previous 6 months, (3) being at least 18 years old, and (4) not deriving their primary income from horse-related services (e.g., training, veterinary care, farriery). The fourth criterion was considered to minimize biases associated with participants possessing atypically extensive education or experience regarding horse health compared to the average horse owner. Eligibility was assessed via an online questionnaire upfront scheduling the study appointment for those who met the criteria.

### 2.3 Data analysis

Upon completion of all study appointments, audio and screen recordings from each session were transcribed manually and independently verified by two researchers to ensure accuracy. Any discrepancies were resolved through mutual cross-checking. The qualitative data (verbalized search thoughts and post-search interviews) were thematically analyzed using an iterative coding approach, where emerging themes related to search strategies, perceived barriers, and decision-making processes were identified. The quantitative data (survey responses and behavioral metrics) were statistically analyzed using descriptive statistics (e.g., means, standard deviations, frequencies) to characterize search behaviors and participant profiles. The classification of participants’ search behavior during the study appointment followed the methodology described by Perez et al. (16). All analyses were conducted using Microsoft Excel.

## 3 Results

### 3.1 Demographics

17 individuals participated in the study, with the sample predominantly composed of females (*n* = 16). Nine participants were aged between 30 and 39 years, four were between 20 and 29 years, and four were between 40 and 70 years. In terms of equestrian disciplines, participants reported engaging in English riding (*n* = 9), Western riding (*n* = 5), carriage driving (*n* = 4), and other disciplines (*n* = 3). Additionally, six participants indicated that they train their horses with a competition-oriented approach. Attachment to their horses, as measured by the LAPS, was high, with the majority of participants (*n* = 14) scoring 50 points or above out of a maximum of 69. Regarding the duration of horse ownership, most participants (*n* = 10) reported owning horses for at least 5 years, with four participants having owned horses for a minimum of 20 years.

Participants recalled that their horses experienced illness with varying frequencies: “every few years” (*n* = 5), “approximately once per year” (*n* = 7), or “roughly every 6 months” (*n* = 5). Self-evaluations of their knowledge about horse health were reported as “neither little nor good knowledge” (*n* = 6), “good knowledge” (*n* = 8), or “very good knowledge” (*n* = 3). Furthermore, only a minority of participants (*n* = 4) appeared not to be anxious about their horses’ health, whereas the majority was classified by the mSHAI as being anxious (*n* = 6) or exhibiting hypochondriac tendencies (*n* = 7). For a complete overview of the participant characteristics see Tables 1, 2.

**Table 1:** Overview of the participants’ demographic characteristics gender, year of birth, equestrian disciplines and duration of horse ownership.

Participant	Gender	Year of birth	Equestrian discipline(s)	Competition-oriented approach?	Years of horse ownership
P_1	F	1979	English riding	No	25
P_2	F	1990	Western riding	No	2
P_3	F	1986	English riding, breeding	No	25
P_4	F	1992	English riding	No	3
P_5	F	1999	Western riding	Yes	6
P_6	F	1999	Western riding	Yes	14
P_7	F	1994	Western riding	No	3
P_8	F	1998	English riding, carriage driving	No	2
P_9	F	1974	Western riding	No	4
P_10	F	1990	English riding	No	1
P_11	M	1971	Carriage driving	Yes	30
P_12	F	1988	English riding, carriage driving	Yes	7
P_13	F	1987	English riding, carriage driving	Yes	4
P_14	F	1996	English riding	No	12
P_15	F	1977	Walks with the horse	No	25
P_16	F	1993	English riding	Yes	10
P_17	F	1994	Ground work, circus lesson, walks with the horse	No	9

**Table 2:** Overview of the participants’ demographic characteristics frequency of experienced horse illnesses, self-assessed horse health knowledge, LAPS score and mSHAI score and category.

Participant	Frequency of horse illness	Horse health knowledge^a^	LAPS^b^	mSHAI^c^
P_1	Approximately once per year	Neither little nor good knowledge	57	14 (not health anxious)
P_2	Approximately once per year	Neither little nor good knowledge	58	28 (hypochondriac)
P_3	Roughly every six months	Very good	60	27 (hypochondriac)
P_4	Roughly every six months	Good	43	24 (health anxious)
P_5	Approximately once per year	Good	66	42 (hypochondriac)
P_6	Every few years	Good	61	18 (health anxious)
P_7	Roughly every six months	Good	54	15 (health anxious)
P_8	Approximately once per year	Neither little nor good knowledge	48	7 (not health anxious)
P_9	Roughly every six months	Neither little nor good knowledge	65	14 (not health anxious)
P_10	Every few years	Neither little nor good knowledge	49	27 (hypochondriac)
P_11	Approximately once per year	Good	51	16 (health anxious)
P_12	Approximately once per year	Good	52	13 (not health anxious)
P_13	Approximately once per year	Very good	58	27 (hypochondriac)
P_14	Every few years	Very good	57	35 (hypochondriac)
P_15	Roughly every six months	Good	61	24 (health anxious)
P_16	Every few years	Good	58	51 (hypochondriac)
P_17	Every few years	Neither little nor good knowledge	65	20 (health anxious)

a Self-assessed on a discrete scale providing the values “no knowledge” – “very little knowledge” – “little knowledge” – “neither little nor good knowledge” – “good knowledge” – “very good knowledge” – “excellent knowledge”.

b Lexington attachment to pets scale.

c Modified short health anxiety inventory.

### 3.2 Reported search behavior from initial interview

When asked about the typical frequency of online searches for equine health information, participants reported a range from “about once a year” (*n* = 3) to “more than once a month” (*n* = 3).

Regarding the context of their most recent online horse health information search (OHHIS), most participants recalled conducting the search either at their horse’s stable (*n* = 8) or during a quiet period at home (*n* = 7). A smaller number reported that their search took place while commuting (*n* = 2) or at work (*n* = 1; one participant reported two recent searches). In terms of device usage, the majority of searches (*n* = 16) were performed on a smartphone, whereas two searches were conducted using a computer. The majority of recalled OHHIS instances occurred prior to a veterinarian consultation (*n* = 14), with the remaining four conducted post-consultation. The objectives of these searches varied: The aims were to gather additional information regarding the horse’s current condition (*n* = 9), to explore preliminary diagnoses (*n* = 6), to obtain information perceived as missing after a veterinary consultation (*n* = 4), to prepare for an upcoming veterinary visit (*n* = 3), and to seek action recommendations (*n* = 1) or assistance in deciding whether veterinary consultation was necessary (*n* = 1).

### 3.3 First thoughts on the horse’s health situation from the video

When asked about their initial impressions and questions after watching the video, the participants expressed uncertainty regarding the horse’s health status (including thoughts about the underlying causes), the severity of the condition, the necessity of contacting a veterinarian or other people for advice, and potential associated costs. Additionally, some participants reported experiencing emotional responses such as pity for the horse (*n* = 1), a desire to cry (*n* = 1), or irritation/uncertainty/concern (*n* = 3).

### 3.4 Task search behavior from screen analysis

For the OHHIS conducted during the study appointment, all participants chose the Google search engine as their starting point. Overall, participants generated an average of 3.71 distinct queries per search session (SD = 2.02). The queries used by participants averaged 3.81 words (SD = 1.57), with the initial query averaging 3.47 words (SD = 1.55). Approximately 29% of the total of 63 queries incorporated multiple descriptors of the horse’s condition (e.g., several symptoms detailing the horse’s current status). About 52% of the queries contained symptom-related information, constituting an evidence-directed search, while nearly 37% included diagnostic terms, reflecting a hypothesis-directed approach. About 11% of queries did reflect an action/treatment seeking approach. From the first queries submitted by each participant, approximately 47% were evidence-directed, and around 41% were hypothesis-directed. Nearly 12% of first queries did reflect an action/treatment seeking approach. Some queries encompassed searches for general information on equine vital signs or specific situations (*n* = 4). Approximately 10% of the queries also included a brief description of the horse’s current state (e.g., while at rest). About 14% of queries articulated a specific search goal (e.g., “diagnosis” or “first aid”). Question-like search terms comprised about 13% of the queries. Regarding search strategies, the largest group of participants employed an analytical-recursive approach (*n* = 6; using both, evidence gathering and hypothesis testing, but no action/treatment seeking), whereas others utilized either a simple (*n* = 4; using only one search strategy) or an intuitive pattern (*n* = 4; using action/treatment seeking before both, evidence gathering and hypothesis testing). Three participants applied an analytical-methodical approach (using both, evidence gathering and hypothesis testing, before action/treatment seeking).

Among the participants, two individuals exclusively read the provided result summaries without clicking on any links. From all participants, a total of 33 search queries did not result in any clicks. An additional three participants refrained from clicking on result links but engaged with the “Additional Questions” section by opening at least one suggested answer. The majority of participants (*n* = 16) limited their examination to the first page of search results, with only one participant navigating to the second page. This individual notably also recorded the highest score on the horse health anxiety scale.

On average, participants clicked on 0.97 results per search query (SD = 1.38). When considering only those queries that resulted in at least one click (*n* = 30), the average number of clicks per query increased to 2.03 (SD = 1.35). The average ranking position of a clicked result was 3.73 (SD = 3.12), thus placed third or fourth in the list of search results. In approximately 72% of cases, the titles of clicked results contained at least one keyword from the search query (excluding filler words and the term “horse”). In about 18% of instances, the result title included a minimum of two keywords from the query (or the keyword if only one was included). In roughly 13% of cases, all keywords from the query were present in the title.

Only one participant’s search involved clicks on results marked as advertisements. The clicked search results were predominantly websites (*n* = 34), with fewer clicks recorded on elements from the “Additional Questions” section (*n* = 15), videos (*n* = 7), forums (*n* = 3), or items in the “Videos” section of the search results (*n* = 2). Most results were authored by feeding manufacturers (*n* = 15), followed by horse veterinary clinics (*n* = 8) and private citizens (*n* = 5).

### 3.5 Task search evaluation from follow-up interview and questionnaires

At the conclusion of the study session, participants were asked to rate their satisfaction with the search process on a 7-point Likert scale, with responses ranging from “unsatisfied” (*n* = 3) to “very satisfied” (*n* = 1). Higher satisfaction ratings were occasionally accompanied by remarks noting that answers were obtained rapidly, whereas lower ratings were often linked to residual uncertainty or a perceived inadequacy in personal search skills.

**Figure 2:**
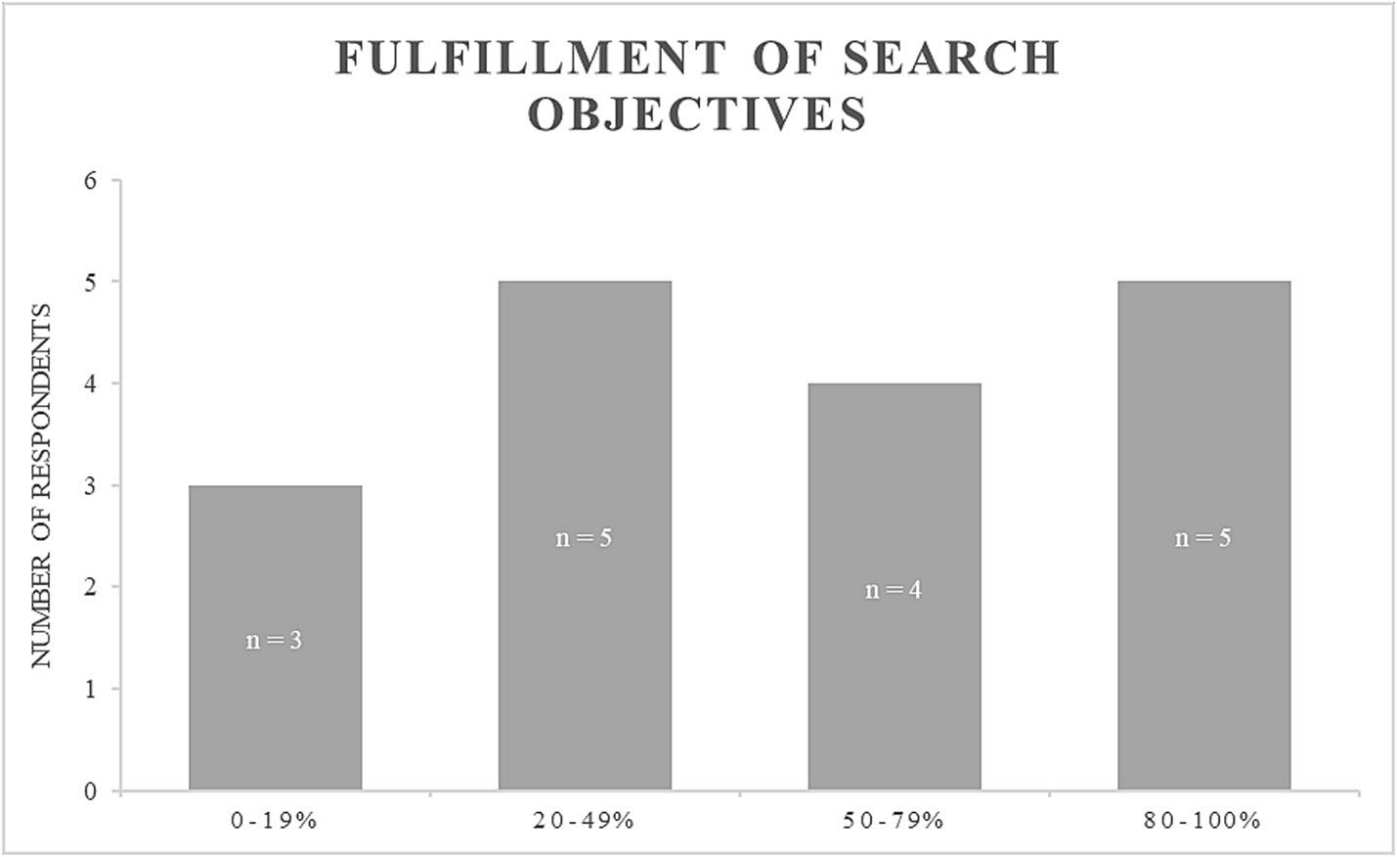
Participants’ assessment of the fulfillment of their search goals.

When summarizing their search outcomes, some participants noted that the search did not significantly assist them in evaluating the situation. The majority tried to provide a preliminary diagnosis or rationale for the horse’s health condition, with suggested explanations including, e.g., throat blockage (*n* = 5), equine asthma (*n* = 2), and pain-associated behavior (*n* = 1). Participants’ assessments of the extent to which their search objectives were fulfilled ranged from 0 to 100% (see Figure 2).

High fulfillment ratings were sometimes associated with comments about the search results being reassuring, while comments on other ratings reiterated a persisting uncertainty and a lack of sufficient input options. Participants identified several elements as necessary for their search experience to be fully satisfactory. Specifically, they indicated that the inclusion of at least one preliminary diagnosis (*n* = 9) and detailed, explained action recommendations (*n* = 9) were essential. Additionally, well-structured, summarized, and easily accessible information was deemed important (*n* = 7), along with guided input regarding the animal and its contextual factors (*n* = 6). Some participants also highlighted the need for the absence of sponsored content (*n* = 4)/the presence of trustworthy websites (*n* = 2), as well as being able to include contextual information (*n* = 3) and receiving assurance that every relevant aspect was considered for the given information (*n* = 2). Moreover, participants’ suggestions included the incorporation of audio recognition of the horse’s sounds (*n* = 1), information on normal vital sign values (*n* = 1), and the provision of visual media (pictures or videos) depicting a horse with similar symptoms (*n* = 1).

When asked regarding the specific objectives of their search, participants reported goals that included obtaining an assessment of urgency (*n* = 7), receiving action recommendations (*n* = 6), obtaining a preliminary diagnosis (*n* = 4), confirming their own assumptions (*n* = 4), preparing for subsequent discussions with a veterinarian (*n* = 3), acquiring background information on the horse’s current condition (*n* = 2), seeking calming (*n* = 2), obtaining decision support (*n* = 1), assessing the need to isolate the horse (*n* = 1), and receiving a detailed action plan (*n* = 1). Following the search, most participants indicated an intention to (re)contact their veterinarian (*n* = 15). Other reported follow-up actions included consulting with peers to exchange opinions and experiences (*n* = 7), further observing the horse (*n* = 6), implementing their own proposed actions (*n* = 3), or adhering to the action recommendations provided by the search results (*n* = 1).

Most participants evaluated their search behavior during the study appointment as “very similar” (*n* = 9) or “similar” (*n* = 7) to their typical search behavior at home. However, some noted that at home they might have used a smartphone rather than a computer, conducted searches iteratively over a longer period (resuming the search as new ideas emerged), or even have chosen not to search the Internet at all. One participant described their behavior as “somehow similar,” adding that at home, the presence of the actual horse and the availability of more time might have influenced the search process.

When queried about obstacles encountered during the search process and suggestions for improvement of the search as a whole, participants identified several areas of concern. Those suggestions included:

- *Source reliability:* Some participants suggested the removal of advertisements (*n* = 4) or pointed out that only trustworthy information sources should be available (*n* = 6). Few participants explicitly wished for access to multiple opinions, including experiences from other horse owners, particularly regarding rare diseases.
- *Action recommendations:* Several participants emphasized the need for rapid delivery of appropriate action recommendations (*n* = 7), such as a specific, followable checklist.
- *Guidance in search process:* A number of participants expressed their desire for more guidance during the search process, e.g., receiving follow-up questions to their input or following an anamnesis questionnaire (*n* = 6). It was suggested that, for ambiguous or hard-to-describe symptoms, it should be possible to select the most suitable media (video, photo, or audio) out of a set of possible alternatives.
- *Contextual information:* Participants expressed a need to incorporate contextual details about the horse and its situation (*n* = 5), allowing for the simultaneous inclusion of multiple symptoms and relevant background information.
- *Automated analysis:* Automated analysis of video, photo, or audio data was suggested by some participants (*n* = 4).
- *Query formulation assistance:* Some participants requested help in identifying appropriate keywords for their searches (*n* = 2) or desired an intuitive search experience that would yield relevant results even when, e.g., describing symptoms in natural language (*n* = 1).
- *Summarized information:* There was a call for information to be presented in a clear and summarized manner (*n* = 3).
- *Preliminary diagnosis:* A few participants indicated a preference for receiving one or multiple preliminary diagnoses (*n* = 2).
- *Efficiency and precision:* Some participants noted the desire for a more efficient search process that would deliver precise results with less effort (*n* = 2).
- *Objective evaluation:* Participants desired the availability of a specialized, objective algorithm for evaluating input (*n* = 2), which would remain unbiased even when user input is formulated to suggest a particular outcome. This should be while also considering the possibility of specific disease etiologies relevant to the individual horse.
- *Language preferences:* Receiving information in the user’s native language was mentioned (*n* = 2).
- *Background information:* Additional background information on the diseases or symptoms (e.g., physiological processes) was desired (*n* = 2).
- *Interactive features:* Some participants expressed a desire for voice/audio interaction capabilities (*n* = 2).
- *Comparison media:* The provision of videos, photos, or audios for comparative purposes was suggested (*n* = 1).
- *Search goal specification:* A feature allowing users to define their search goals at the outset was proposed (*n* = 1).
- *Symptom selection:* The option to select symptoms from a predefined list was desired (*n* = 1).
- *Veterinarian availability:* Information regarding the (digital or physical) availability of veterinarians was considered to be useful (*n* = 1).
- *Streamlined access:* Participants also suggested eliminating the need to remember login details for, e.g., websites (*n* = 1) and reducing the requirement to open multiple tabs to save information (*n* = 1).

## 4 Discussion

### 4.1 Principal results

**Figure 3:**
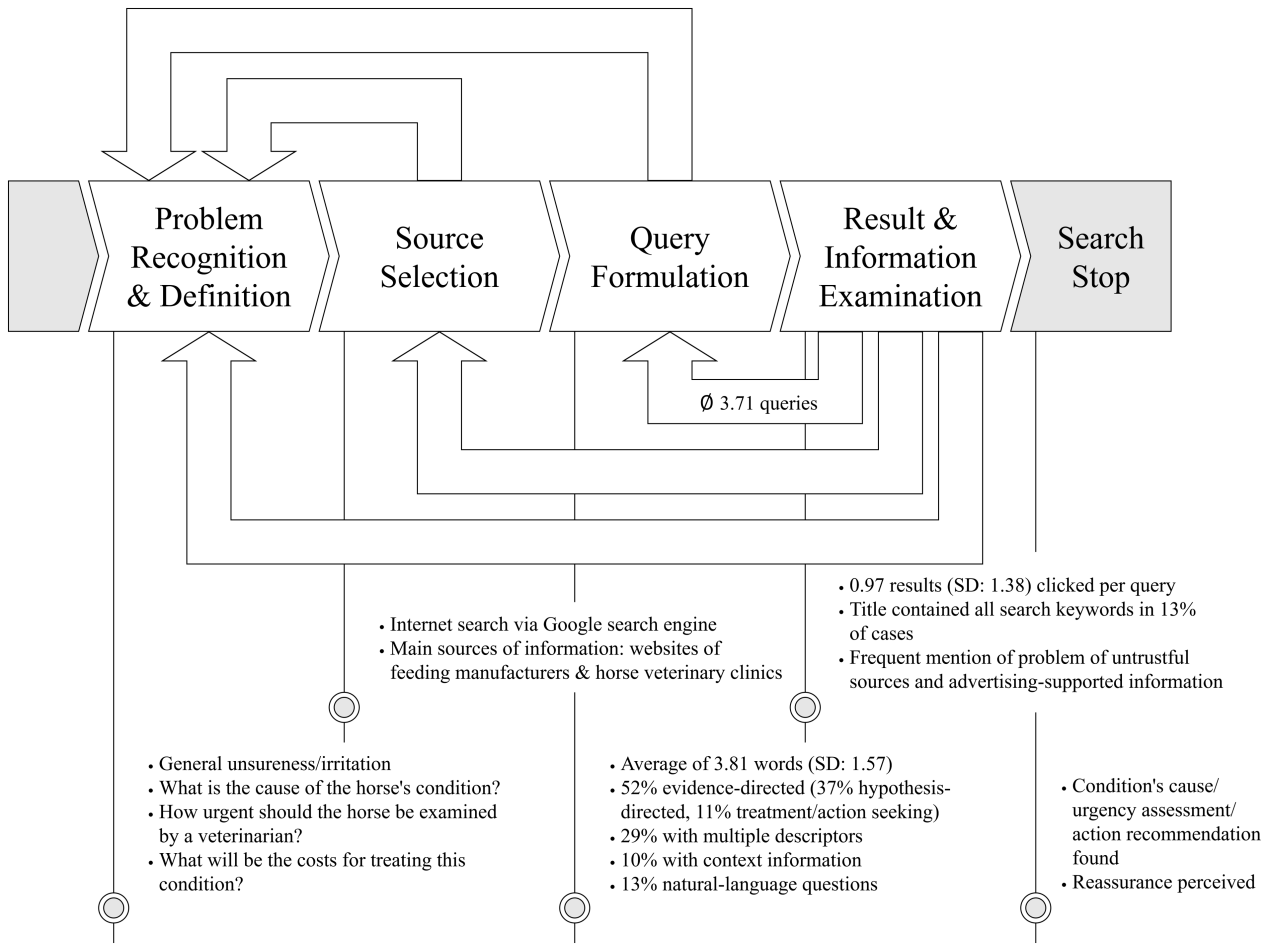
Summary of information gained from the study on the participants’ horse health information seeking process.

This study aimed to examine horse owners’ search behavior and associated information needs. Unlike previous research that presented participants with textual test vignettes – prompting searches based on provided symptom descriptions (19, 23) – this study employed a video to present the scenario, thereby potentially enhancing the ecological validity of the observed information behavior. Figure 3 illustrates the key information for each stage of the information-seeking process. Participants indicated that improvements in their search experience could be achieved through assistance in formulating precise search queries, guidance throughout the search process, and automated analysis of multimedia inputs such as pictures, videos, and audio recordings.

### 4.2 Limitations

The general population of equestrians in Germany shows an average age of about 38 years and consists of 76% female equestrians (22). Compared to that, the sample in this study was slightly younger, although no underage individuals participated, and there was a notable gender imbalance with only one male participant out of seventeen. Additionally, not all equestrian disciplines were represented; for instance, vaulting [practiced by about 15% of equestrians (22)] was absent in the sample, while carriage driving was overrepresented [practiced by 24% in the sample and by 9% of the general population of equestrians in Germany (22)].

Although the sample included individuals with varying lengths of horse ownership, the overall level of experience may be higher than that of equestrians in general (as equestrians usually purchase horses only after they have gained several years of experience with horses), but may be typical for horse owners. The sample consisted of horse owners who demonstrated a high level of attachment to their horses; a characteristic typically observed among actively engaged equestrians (22). Moreover, the proportion of horse owners employing a competition-oriented training approach was comparable to that of active equestrians affiliated with equestrian associations.

The study environment was controlled and surveilled, which might have influenced participant behavior. While the interview data suggested that participants’ search strategies generally resembled their typical behavior, some participants reported feeling time pressure, indicated that their search approach might differ when using another device (e.g., a mobile phone), or noted that their actions could have been altered by the absence of their own horse and the real-life context. Despite these potential discrepancies, the use of a video to simulate the search scenario represents a reasonable compromise for eliciting realistic behavior (23).

Furthermore, self-reported search behaviors, including details of the search situation, frequency, and device used, might have been subject to recall bias, with atypical search experiences possibly being overemphasized in participants’ recollections (24). The specification of information needs during the search process is inherently challenging, as these needs are dynamic and can change over time (25). To address this, participants were only queried about their search goals at the end of the search and the result summary was additionally taken into account, thereby capturing the most salient aspects in hindsight.

For future research, the incorporation of eye-tracking methods could enhance the evaluation of the factors influencing the selection of clicked results, offering a more detailed understanding of users’ attention and decision-making processes during online searches. Also, while other types of animal owners appear to have similar characteristics in relevant aspects (5), additional research should be conducted for those animal owners to verify the obtained results for other contexts. Due to differences in animal health insurance coverage (8), different types of animal owners may use different levels of search before contacting a veterinarian.

### 4.3 Comparison with prior work

The present study contributes to a relatively underexplored area by examining the search behavior of (pet) animal owners, respectively horse owners.

One of the observations from our study was a strong emotional reaction of some participants to a possible illness of their horse. As already discussed by Haase (5), (pet) animal owners might be in an emotionally tense state when they consider their animal to be ill, which might influence their ability to critically assess and process health-related information.

Our findings indicate that the choice of search device differs between parents and horse owners conducting proxy health searches. In a prior study on parental online health-seeking behavior, 81% of participants used a computer (26), whereas only 11% of horse owners in our study reported to do so. Instead, reported smartphone usage was significantly higher among horse owners (81%) compared to parents (63%) (26). This discrepancy could be attributed to the context in which searches take place – parents often search from home or work, where desktop computers or laptops are more accessible, whereas horse owners may be at the stable or outdoors, where a smartphone is the more practical option (5). The variance could also be the result of a timely development since the publication of the parent-related study, going hand in hand with a more frequent use of smartphones instead of computers (probably also for research activities) (27–29).

Another key difference between the two groups lies in the timing of searches relative to professional consultations. A previous study has shown that 95% of parents searched for information after visiting a doctor and 63% were searching beforehand (26), whereas in our study, only 24% of horse owners reported searching after a veterinary consultation, with the majority (76%) conducting searches beforehand. Although, in contrast to the study design of Yardi et al. (26), our study design required participants to select only one specific search event, these findings hint at underlying differences in the pre- and post-consultation information needs of parents versus horse owners. The findings suggest that horse owners are more likely to use online resources to assess symptoms and determine whether a veterinary visit is necessary, whereas parents may use online searches primarily to supplement or verify medical advice they have already received. This distinction might highlight the greater financial and logistical constraints associated with veterinary care, where animal owners may be hesitant to incur costs for a consultation without first attempting to self-assess the severity of or self-handle their animal’s condition (5, 30).

The search objectives of both groups show notable similarities, as both parents and horse owners primarily seek to (1) obtain a preliminary diagnosis, (2) assess the severity of the condition, and (3) determine whether professional consultation is necessary (26, 31). These goals align with the fundamental motivation behind proxy health searches: reducing uncertainty and gathering information (e.g., to make decisions) (32). It is also noteworthy that, irrespective of the user group, the Google search engine remains a predominant tool for accessing health and veterinary information – a trend that has been consistently observed across various studies (31, 33, 34).

However, differences emerge in search strategies. Our study revealed that horse owners were more likely to conduct their first search attempt as hypothesis-directed searches (41%) compared to parents (4%) (33), meaning they searched for specific diseases rather than relying solely on symptom descriptions more often. Conversely, a lower percentage of horse owners (47%) initiated their search using evidence-directed approaches compared to parents (87%), meaning the latter started their search more often by looking for symptom descriptions. About 12% of the horse owners in our study began their search by looking for action/treatment options, whereas 9% of parents in previous studies did so (33). These patterns suggest that horse owners focus more on identifying the correct diagnosis before considering treatment, possibly due to the higher risk of misinterpreting symptoms in animals compared to humans (due to their different physiology).

The analysis indicated that horse owners were more inclined to adopt a simple search strategy (24%; using only one search strategy out of hypothesis testing, evidence gathering and action/treatment seeking) than a self-seeker for online health information (0%) (16). However, both groups predominantly utilized an analytical searching strategy (80% of self-seekers and 53% of horse owners; using both, hypothesis testing and evidence gathering before, if applicable, action/treatment seeking) (16).

Despite these strategic differences, the initial search queries of the group of parental proxy seekers and horse-owning proxy seekers were remarkably similar in length, as evidenced by the nearly identical average number of words used (3.77 for parental seekers versus 3.47 for horse owners) (33). Parts of both groups also tended to include contextual information in their queries; parents sometimes included descriptors such as “adolescent” to refine the search engine’s output (31, 33), and a comparable approach to context-inclusion was observed among some horse owners. Finally, the overall search process differed in terms of query iteration: horse owners submitted, on average, one additional query compared to parents (3.71 versus 2.95 queries of parental by-proxy seekers) (33). This may indicate that horse owners engage in a more extended search process, potentially reflecting a need for additional information to resolve uncertainties as hypothesized by Haase (5).

The average position of the clicked search results was distinctly higher in the result list for parental by-proxy seekers [mean position 2.4 (33)] compared to horse owners (mean position 3.73). This finding suggests that parents may be more inclined to select results from the top ranks of search engine listings, potentially reflecting a higher trust in the algorithms that prioritize these links or a more focused search intent.

In terms of browsing beyond the first page of search results, a similarly small proportion of people from both groups was observed, with 6% of horse owners and 8% (31), respectively 13% (33) of parents venturing into subsequent pages.

When examining the types of websites accessed, a divergence in preferences emerged. Prior research has shown that the general population typically favors websites associated with medical doctors, hospitals, and nursing services for health-related searches (1). Consistent with this pattern, horse owners included sources associated with health-care professionals among their selections. For horse owners, feeding manufacturers were the most frequently accessed source of information. In searches related to human health, food or dietary supplement manufacturers were also commonly used, but only as the most frequently accessed non-medical source (1). This divergence might be due to differences in the availability of information sources in each of the domains.

Furthermore, the usage of online forums slightly differed between the different contexts. For parental by-proxy seekers, a higher rate of forum utilization (29%) is reported (26) compared to horse owners (18%). The usage is similar when compared to self-seekers for online health information, which is reported at 17% (1). The role of forum information in shaping health-related decisions remains contentious in the literature, with some studies affirming its reliability and others cautioning against potential quality issues (3).

Finally, the issue of trust in online health information was a major concern for both groups. Studies on parental searches reveal that many parents struggle to assess the reliability of online medical information, leading to uncertainty about the credibility of their findings (26). Our study suggests that horse owners experience similar concerns, particularly regarding the lack of transparent quality indicators in search results. This aligns with broader research on the challenges of applying online health information to real-life situations, particularly in cases where laypersons must navigate complex medical terminology and conflicting advice (35).

Overall, our findings suggest that horse owners, as a subgroup of proxy health seekers, exhibit both shared and unique search behaviors compared to parents.

## 5 Conclusion

This study provides new insights into proxy health information-seeking behavior through the specific lens of horse owners searching for veterinary information online. Given the unique context, where the individual affected by a condition is unable to communicate and the by-proxy seeker is unable to transfer personal knowledge, the findings underscore the challenges typically encountered in layperson searches. While this study focuses on horse owners, the findings have broader implications for other proxy seekers, such as other (pet) animal owners, parents or caregivers searching for health-related information. Enhancing online health information systems to better accommodate proxy decision-makers could improve health outcomes for both humans and animals alike. Veterinarians can use the information obtained through this study to better address their clients’ communication needs, provide them with the skills and knowledge they need to conduct online searches, and understand why and how their clients address potential health issues in their animals. This allows them to build a better partnership with them in the area of animal health.

## Data Availability

The raw data supporting the conclusions of this article will be made available by the authors, without undue reservation.
